# Supplementary Motor Area and Superior Parietal Lobule Restore Sensory Facilitation Prior to Stepping When a Decrease of Afferent Inputs Occurs

**DOI:** 10.3389/fneur.2018.01132

**Published:** 2019-01-04

**Authors:** Olivia Lhomond, Normand Teasdale, Martin Simoneau, Laurence Mouchnino

**Affiliations:** ^1^Aix Marseille Univ, CNRS, Laboratoire de Neurosciences Cognitives, Marseille, France; ^2^Faculté de médecine, Département de kinésiologie, Université Laval, Québec, QC, Canada; ^3^Centre Interdisciplinaire de Recherche en Réadaptation et Intégration Sociale, Québec, QC, Canada

**Keywords:** step movement, somatosensory evoked potential, body representation in brain, supplementary motor area (SMA), balance control

## Abstract

The weighting of the sensory inputs is not uniform during movement preparation and execution. For instance, a transient increase in the transmission to the cortical level of cutaneous input ~700 ms was observed before participants initiated a step forward. The sensory facilitation occurred at a time when feet cutaneous information is critical for setting the forces to be exerted onto the ground to shift the center of mass toward the supporting side prior to foot-off. Despite clear evidence of task-dependent modulation of the early somatosensory signal transmission, the neural mechanisms are mainly unknown. One hypothesis suggests that during movement preparation the premotor cortex and specifically the supplementary motor area (SMA) can be the source of an efferent signal that facilitates the somatosensory processes irrespectively of the amount of sensory inputs arriving at the somatosensory areas. Here, we depressed mechanically the plantar sole cutaneous transmission by increasing pressure under the feet by adding an extra body weight to test whether the task-dependent modulation is present during step preparation. Results showed upregulation of the neural response to tactile stimulation in the extra-weight condition during the stepping preparation whereas depressed neural response was still observed in standing condition. Source localization indicated the SMA and to a lesser extent the superior parietal lobule (SPL) areas as the likely origin of the response modulation. Upregulating cutaneous inputs (when mechanically depressed) at an early stage by efferent signals from the motor system could be an attempt to restore the level of sensory afferents to make it suitable for setting the anticipatory adjustments prior to step initiation.

## Introduction

Anticipatory postural adjustments (APAs) precede different voluntary lower limb movements [leg flexion: (1, 2); lateral leg raising: (3); gait initiation: (4, 5). For example, in gait initiation, the leg movement is always preceded by a shift of the center of mass (CoM) toward the supporting side and forward to create the condition for proper step movement execution. Part of these APAs are aimed at unloading the leg to be moved and preserving balance during the movement. It has been demonstrated by Massion (6) that the APAs are centrally preprogrammed and prepared from at least 1,400 ms before step execution as reported by Mackinnon et al. (7). During gait initiation, monitoring the initial standing condition is a prerequisite for setting of the APAs [e. g., (8–11)]. For instance, Timmann and Horak (10) showed that the anticipatory phase that propels the body forward is reduced when a backward platform displacement is triggered during the planning phase of the stepping movement. This suggests that sensory inputs regarding the new standing conditions are controlled online and can be rapidly processed to alter the APAs based on visual, vestibular, proprioceptive, tactile information related to body current position relative to the support. Among the sensory receptors that convey information concerning balance, plantar sole tactile receptors are well suited to detect the mere transient changes in the contact forces between the feet and the ground to alter the forthcoming APAs. For example in the absence of any vestibular and visual inputs, the amplitude of the APAs is changed according to the current body position in space on the basis of cutaneous cues with at least some contribution of proprioceptive information (12). In addition, Lin and Yang (13) showed a decrease of the mediolateral APAs after desensitization of the plantar sole cutaneous receptors by immersion in cold water; the greater the desensitization the smaller the APAs amplitude. This is not the case, however, when some sensory inputs remained from one of the feet (14), that is, after unilateral tibial nerve block. Altogether, these results indicate that plantar cutaneous and intrinsic foot muscle proprioceptive inputs (15) provide information for shaping the centrally programmed APAs.

In support of these behavioral studies, we have recently demonstrated using electrophysiological techniques, that the early transmission of cutaneous inputs from the periphery to the cortex was facilitated during the planning phase of gait initiation [about 700 ms before any muscular activity for motor execution, (16)]. Such variation was observed as early as 55 ms after an electrical stimulation of the cutaneous receptors of the plantar sole (16) or the fingers (17). This observation was interpreted as reflecting the activity of the primary somatosensory cortex (SI) (18–20). These authors and others [for example, (21)] have shown that this early sensory process is related to the incoming sensory inputs and is representative of the stimulus characteristics (e.g., intensity, frequency). In addition, Duysens at al. (22) have reported an increase of the perception of tactile stimuli when sensory transmission is increased. Therefore, the sensitivity of the sensory cortex to afferents is supported by an attenuation or a facilitation of the somatosensory evoked potentials (22, 23). A “task-related facilitation” mechanism might therefore contribute to enhance perception of tactile inputs when sensory information is relevant for performing the task. This is in line with Bolton et al.'s study (24) which demonstrated that when the somatosensory information coming from the hand is used to control balance, the somatosensory evoked potential following the median nerve stimulation is increased. This process referred to as “task-related sensory facilitation” presumably serves to optimize the monitoring of equilibrium during quiet upright standing (24) as well as during the planning phase of gait initiation (16). During movement preparation the premotor cortex and specifically the supplementary motor area (SMA) can be the source of an efferent signal prompting sensory facilitation. Indeed, during movement preparation, various authors have observed a specific preparatory cortical activity known as the movement-related contingent negative variation [CNV; (25–28)]. During the final stage of the CNV, an increase in the SMA activity was reported (25) possibly to set the APAs timing (29). In addition, an increase activity of the SPL (an important node for sensorimotor integration) was noted when somatosensory afferents were stimulated (30). The SMA is recognized to have direct connections with the sensorimotor cortex (31, 32) and is also interconnected to the SPL (33). The link from the SMA to the sensory mechanism can be indirectly revealed by source localization analysis. For instance, activation of the SMA and pre-motor areas were time-locked to somatosensory facilitation following tactile stimulation (16). Such increase in the activation of the SMA was also observed when the demands of locomotor tasks require increased processing of sensory information even when the tasks were imagined [imagining walking, initiating gait, walking with obstacles, (34)].

Despite clear evidence of the effect of sensorimotor tasks on the response to cutaneous stimulation, the neural mechanisms underlying sensory facilitation are mainly unknown. One hypothesis proposes that the responsiveness of afferent nerve is increased at spinal level to improve the transmission of information to the supraspinal center (35). Alternatively, the specific facilitation of the response might be evoked by an efferent signal from premotor areas. This is in line with the SMA modulatory function of somatosensory activity used by other cortical areas during self-generated movements (36).

In the present study, we developed a paradigm to determine if motor preparation can evoke a cortico-cortical facilitation during the planning phase of the stepping movement even when the amount of plantar sole afferent is attenuated. To do so, the afferent input from the plantar sole cutaneous mechanoreceptors were decreased by having participants wearing a 20 kg weighted-vest (37). We compared the somatosensory-evoked potentials (SEPs) of healthy participants during upright standing or the preparation of a stepping movement. We expected the SEPs to be larger during the planning phase of the stepping movement in the extra-weight condition.

## Methods

**Figure 1:**
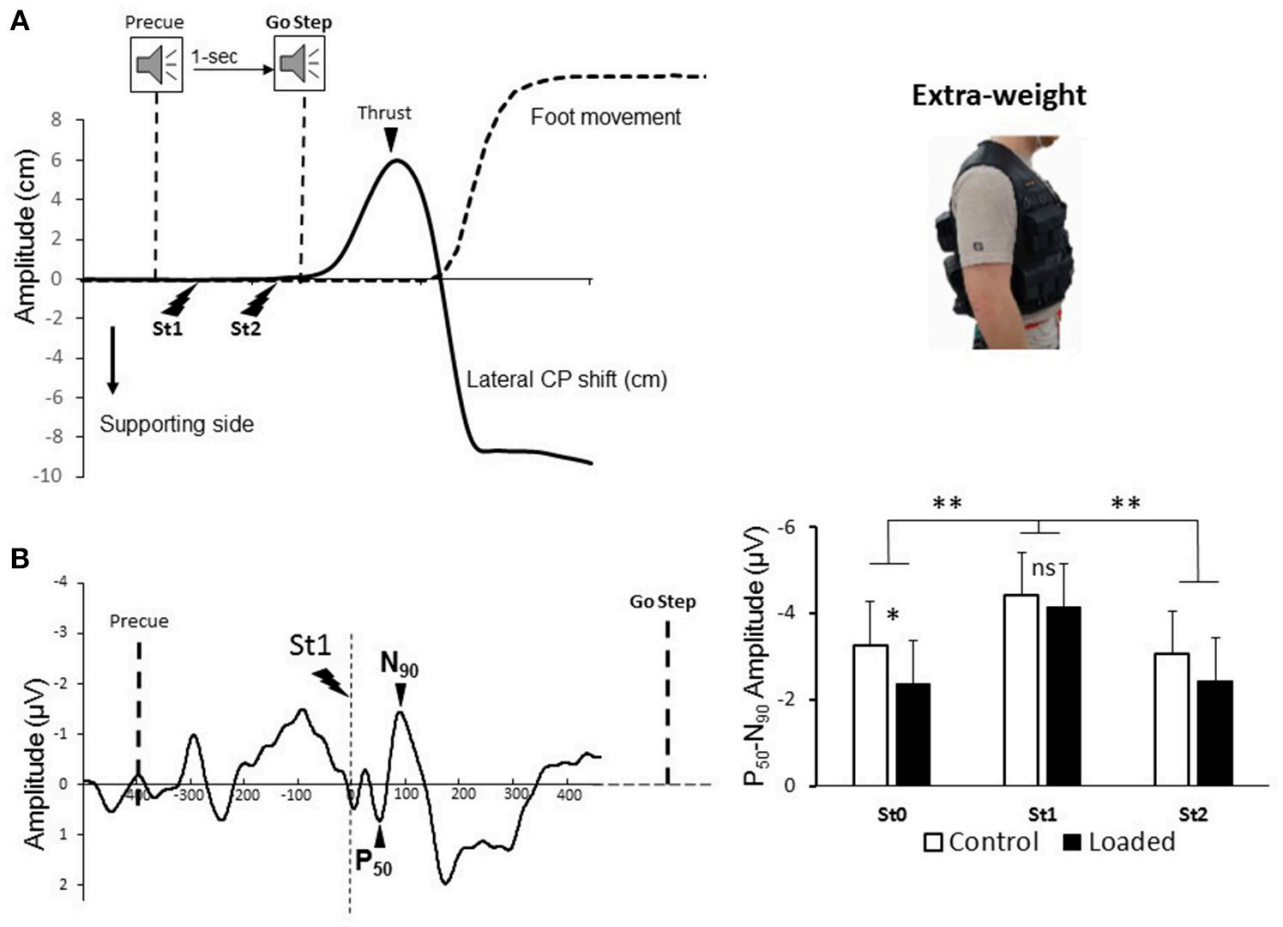
(**A**, left panel) Behavioral recordings during the Stepping task for one representative participant: mediolateral center of pressure, and foot movement in dotted line. Electric stimulations during motor preparation was identified. (**A**, right panel) The 20 kg weight was distributed on the front and back of the vest. (**B**, left panel) Grand average somatosensory-evoked potential (SEP). Dashed line indicates the moment of the stimulation. Note that the first potential observed −300 ms prior to the electrical stimulus was an auditory potential evoked by the pre-cue tone. (**B**, right panel) Mean amplitude for 15 participants of the averaged P_50_-N_90_ SEP evoked by the electrical stimulation and recorded over Cz electrode during Standing (St0) and movement preparation (St1 and St2). (^*^p < 0.05 and ^**^p < 0.01).

Fifteen healthy participants performed the experiment [8 male, mean age: 25 ± 3 years; mean body mass index (BMI): 23.9 ± 2.9 kg/m^2^]. Informed consent was obtained from all participants, and all procedures were approved by the Ethics Committee at Laval University. In the task hereafter referred to as the Stepping task, participants were instructed to step forward with the right leg in response to an auditory signal (a 100-ms tone) keeping their eyes closed (Figure 1A). This auditory Go step signal was preceded 1 s earlier with a pre-cueing tone. This pre-cue signal served as a warning stimulus allowing participants to have a period of preparation (7). During the stepping task, the plantar sole of the forthcoming supporting foot was stimulated twice during the preparation phase of the step that is 600 ms (early preparation, St1) and 100 ms (late preparation, St2) before the Go step signal (see below for the stimulation technique). A control task (hereafter referred to as the Standing task) was performed with a similar design (i.e., 2 auditory signals and 2 electrical stimulations, St0) where participants adopted an upright quiet standing position. In both tasks the participants were standing upright and loading symmetrical. At the start of a task, the participants looked at a fixation point positioned at eye level, ~2 m directly in front of them. One second before the pre-cue signal, participants were asked to close their eyes and receive verbal instruction on the nature of the upcoming task. The Standing and Stepping tasks were randomly presented across the experimental session to prevent preparation of a stepping movement long before task instruction. No more than 2 Standing trials were presented in succession. For both tasks, the same sequence of two tones and two stimulations as in the Stepping task were delivered.

Each participant performed 50 stepping movements (i.e., 100 stimulations). In the Standing task 50 stimulations were delivered. Participants were asked to stand quietly in two conditions: (i) Loaded, participants were standing while wearing a 20-kg weight-vest representing an increased weight of 25 ± 4% (Figure 1A, right panel) and (ii) Control, without extra-weight.

### Stimulation Procedure

The electrical stimulus was delivered by an isolated bipolar constant current stimulator (DS5 Digitimer, Welwyn Garden City, UK). On the supporting foot, the cathode was located under the metatarsal region and the anode underneath the heel (5 × 9 cm electrodes, Platinium Foam Electrodes). The stimulation consisted of a single rectangular 10-ms pulse (16, 37). The stimulation intensity was set to avoid any cutaneous reflexes. The electrical stimulation of the plantar sole activates all nerve fibers associated with the mechanoreceptors including free nerve endings. These mechanoreceptors respond to mechanical skin deformation while electrical stimulus rather activates all the sensory nerves in absence of skin deformation. Due to the position of the electrodes and direction of the current flow between the electrodes, the sensation did not mimic displacement in center of pressure, that is a mechanical stimulation. For instance, the electrical stimulation did not evoke a specific percept of pressure change on the foot plantar sole leading to a postural reaction. For each participant, while in a quiet upright standing position, we determined the lowest intensity leading to constant perception of the stimulation (mean amplitude of 6.9 ± 1 mA). This stimulation was determined as the baseline value. For each participant, the stimulation intensity was set at 25% higher than their perceptual threshold value (but below their motor threshold). The interval between each electrical stimulus was designed to avoid the “interference phenomenon” which is a reduction of the somatosensory evoked potentials when two stimulations are too close in time (38). An interval longer than 300 ms would be sufficient to avoid the interference phenomenon according to Morita et al. (39).

### Behavioral Recordings and Analyses

Ground reaction forces and moments were recorded at a sampling rate of 1,000 Hz through a force platform (AMTI model OR-6-6, Watertown, MA, USA). The APAs were measured by computing lateral center of pressure (CP) (Figure 1A, left panel). First, the lateral CP shift is directed toward the side of the stepping movement: this corresponds to a vigorous thrust onto the ground exerted mainly by the forthcoming moving leg while still on the ground (6). This force shifts the center of mass toward the supporting side to unload the leg performing the stepping movement. After removing the mean of the signal (computed during 1 s from the recording onset), we computed the mean of the trials of each participant and condition. The amplitude of the thrust was defined as the difference between the initial position and the peak toward the stepping side. An electromagnetic sensor located on the top of the right foot recorded the kinematics of the stepping movements (sampling frequency 100 Hz, model Flock of Birds, Ascension Technology Corporation, VT, USA).

### Electroencephalography Recordings and Analyses

Participants were fitted with an EEG system (Geodesic 64-channel EEG sensor net GSN64; Electrical Geodesics Inc., Eugene) sampled at 1,000 Hz. The electrodes were referenced to the vertex (Cz), and then re-referenced to the net average. Data pre-processing was performed with BrainVision Analyzer 2 (Brain Products, Germany). The EEG signals were filtered off-line with 45 Hz (high cut-off) filters (digital filters, 24 dB/octave) and 0.1 Hz (low cut-off) filters (digital filters, 12 dB/octave). Vertical electrodes were recorded bipolarly with electrodes placed above and below the left eye; horizontal electrodes were recorded bipolarly with electrodes positioned near the outer canthus of each eye. The EEG signals were corrected for eye blinking according to the statistical method of Gratton et al. (40).

Somatosensory evoked potentials (SEPs, Figure 1B) were obtained by averaging, for each participant and condition, all synchronized epochs relative to the electrical stimulus. The average amplitude of the 60-ms pre-stimulus epoch served as a baseline. We measured the SEPs over the Cz electrode as this electrode overlays the sensorimotor cortices and, on the homunculus, the feet are located on the inner surface of the longitudinal fissure. The earliest discernible positive (P_50_) and negative (N_90_) peaks after each stimulus were identified. These peak latencies are comparable to latencies measured by Duysens et al. (22) and Altenmüller et al. (23) evoked by stimulating the sural nerve. The fact that the sural nerve is mainly a cutaneous nerve (41) suggests that P_50_-N_90_ originates from cutaneous input. The amplitude of the P_50_-N_90_ waveform was measured peak-to-peak (Figure 1B, left panel).

To estimate the neural sources of the SEPs, we used dynamic statistical parametric mapping (dSPM) implemented in the Brainstorm software [(42), freely available at http://neuroimage.usc.edu/brainstorm]. We used the data from all sensors processed and averaged for each participant, condition and electrode. The forward model was computed using a 3D-shell sphere boundary element model (BEM) on the anatomical MRI brain MNI Colin27 template (15,000 vertices), a predominant volume conductor model (43, 44). The cortical sources were analyzed during 2-time windows that encompass and follow the P_50_-N_90_ SEP (i.e., [50–90 ms] and [90–130 ms]) to find the source of the facilitation observed during motor preparation.

### Statistical Analyses

The SEPs amplitude and latencies recorded at Cz were submitted to repeated measures analysis of variance (ANOVA) with condition (i.e., Loaded and Control) and epoch (St0, St1, and St2) as factors. *Post-hoc* analysis was performed through Newmann–Keuls test. The Standing task was included as a level (i.e., St0) along with St1 and St2 epochs of the Stepping task in a one-way ANOVA. We also conducted paired *t-*test for the statistical source estimation maps for contrasts (i.e., Stepping minus Standing tasks). The behavioral data (i.e., step kinematics and forces) were analyzed using paired *t-*test. All dependent variables (EEG and behavioral data) showed normal distributions (i.e., *P*s > 0.05, Kolmogorov–Smirnov test). The level of significance was set at 5% for all analyses.

## Results

### Somatosensory Evoked Potential

The results for the amplitude of the P_50_-N_90_ SEPs showed a main effect of epoch [Figure 1B right panel, *F*_(2, 28)_ = 9; *p* < 0.001]. The amplitude of the P_50_-N_90_ SEPs was greater during the early preparation of the stepping movement (i.e., epoch St1) than during standing (St0) or late preparation of the stepping movement (i.e., epoch St2). The amplitude of the P_50_-N_90_ SEPs was also altered by the loading [*F*_(1, 14)_ = 4.88; *p* < 0.05]. This attenuation was due to the standing condition [St0, *t*_(14)_ = −2.4; *p* = 0.02] as previously reported by Lhomond et al. (37). It is worth noting, however, that the amplitude of the SEPs was similar in the loaded and control conditions during the early preparation [St1, *t*_(14)_ = −0.66; *p* = 0.51] of the stepping movement. This result suggests that despite sensory attenuation during the standing epoch, it seems that neural mechanisms related to stepping movement preparation alleviate sensory attenuation probably to ascertain proficient APAs. Overall the latencies of the P_50_ did not differ with loading [*F*_(1, 14)_ = 1.14; *p* = 0.3]. The latencies were slightly longer during the early and late epochs of stepping movement preparation (overall means of St1 and St2: 55 ± 11 ms) than for the standing epoch (st0) (overall mean of St0: 50 ± 9 ms) [*F*_(2, 28)_ = 4.8; *p* = 0.015].

### Source Localization

**Figure 2:**
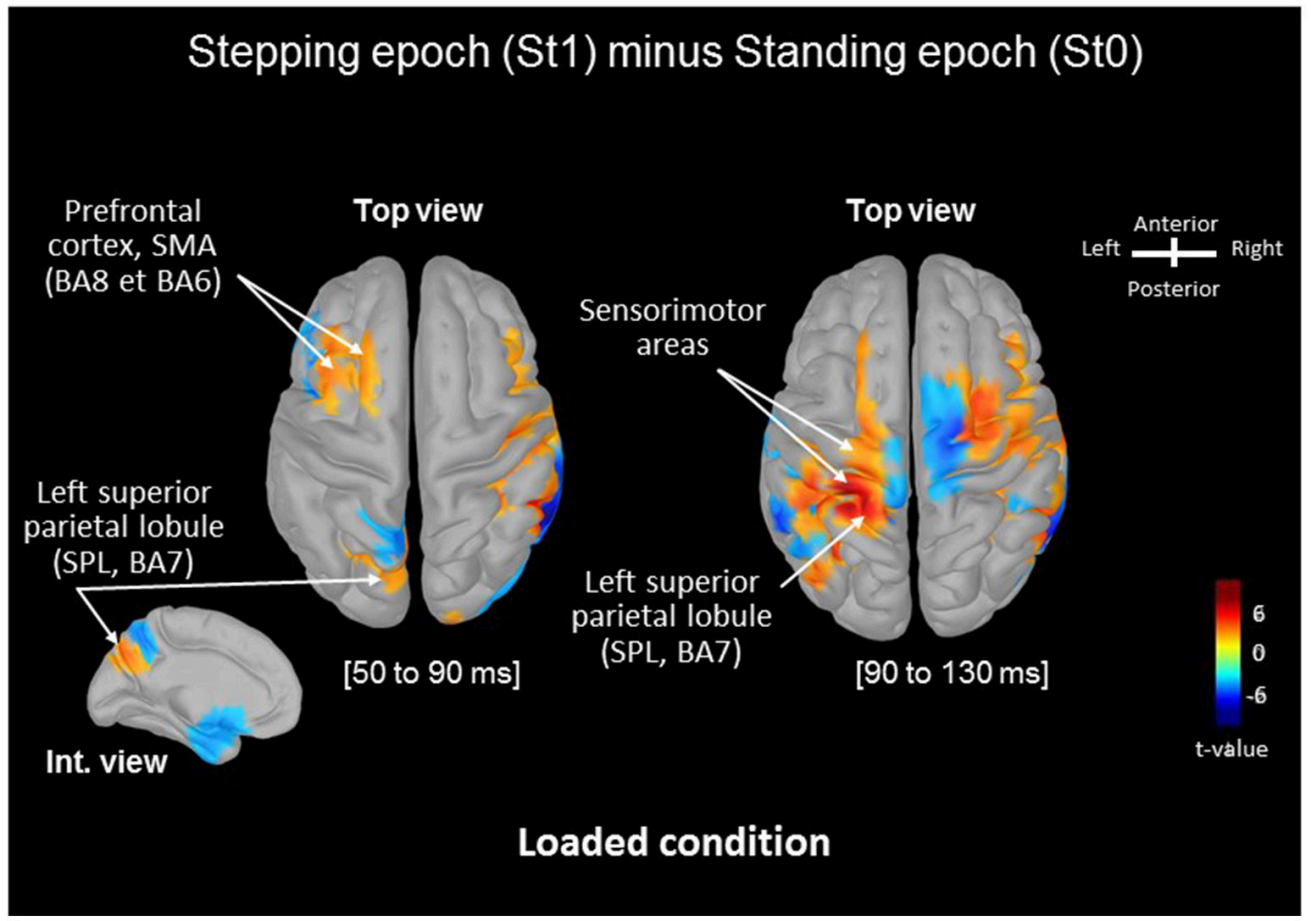
Statistical source estimation maps for St1vs. St0 contrast (i.e., Stepping minus Standing tasks) in the Loaded condition. Significant *t*-values (*p* ≤ 0.05, *n* = 15) of the source localization during the [50–90 ms] and [90–130 ms] time windows projected on a cortical template (MNI's Colin 27). For both windows we display the top cortical view and the internal view of the left hemisphere for the P_50_-N_90_ SEP. The red color represents a higher activity in St1 relative to St0.

Source analysis localized SMA and superior parietal lobule (SPL) as the generators of the increase in amplitude of the P_50_-N_90_ SEPs observed in the early preparation of the stepping movement (St1) in loading condition. Significant differences between the absolute mean activity computed in the Loaded condition is depicted in Figure 2. Starting during the P_50_-N_90_ period [50–90 ms], the SPL shows greater activity during the later temporal window [90–130 ms] during stepping preparation than when solely standing still. We observed marked significant increase in the activation of the primary sensorimotor areas in the later temporal window [90–130 ms].

### Behavioral Results During the APAs

Variables related to motor execution (Stepping movement) were analyzed to verify whether the APAs (i.e., latency, amplitude, duration) varied across the conditions. The APAs onset occurred, on average 120 ± 50 ms after the Go step signal and this value did not differ between conditions [*t*_(14)_ = 1.48; *p* = 0.15]. This short latency attests that participants attempted to synchronize step onset with the Go signal and did not react to it. To further test whether modifications of the APAs occurred due to loading, we analyzed the duration and amplitude of the CP thrust (Figure 1A). The results showed that thrust duration is unchanged by loading [overall mean: 314 ± 31 ms; *t*_(14)_ = 1.25; *p* = 0.22]. The maximal amplitude of the thrust, however, was smaller in the loaded condition [means of 3.7 ± 1.1 and 4.2 ± 1.2 cm for the loaded and control condition, *t*_(14)_ = 3.66; *p* = 0.002]. This result indicates that the amplitude of the APAs is altered according to the loading condition.

## Discussion

By adding an extra-weight on the body to increase the pressure on the plantar sole mechanoreceptors, we have shown that the neural response to the same somatosensory stimulus evoked a decrease of the early P_50_-N_90_ neural response when standing still and an increase of this neural response when preparing for a stepping movement. In the loaded condition during standing, the fact that the P_50_-N_90_ neural response to the stimulation was decreased is consistent with the hypothesis of a depressed transmission of cutaneous inputs arriving at the cortical level (37). This is likely the result of an increase pressure of the foot plantar sole where the mechanoreceptors are embedded (15, 45–49). By contrast, when preparing for a stepping movement the depressed transmission did not prevent the facilitation of sensory processing to occur. This upregulation is consistent with the hypothesis of an efferent signal coming from the premotor areas. The SMA and to a lesser extent the superior parietal lobule (SPL) areas are the likely sources of sensory processing facilitation.

During the early preparation of the stepping movement (i.e., some 720 ms before the APAs execution), efferent signals from the frontal cortex could restore a certain level of sensory processing to ascertain proficient setting of the APAs prior to step initiation. Indeed, motor preparation of the transition from stance to stepping movement requires an estimation of the body's orientation relative to gravity (50). Although several sensors contribute to that “prior knowledge” of body orientation, it can be determined from foot plantar sole cutaneous receptors and intrinsic foot muscle proprioception in absence of visual, vestibular or proprioceptive inputs (12, 15). Depressed afferents reaching the cortical level may have prompted the SMA to provide an efferent signal to the somatosensory regions (31, 32, 36). This is supported by the fact that SMA neurones are sensitive to somatosensory stimuli (here depressed) (51) and that SMA is connected to SI [with no direct connection from the thalamus, for a review (52)]. These interconnections to SI are compatible with the idea that this area may in turn have facilitated the sensory processing during the early motor preparation. In line with this suggestion is the fact that the SMA is known to be activated specifically during movement preparation as it has been reported in studies assessing cortical network related to motor imagery (34, 53). It has been suggested, by Jeannerod (54), that motor imagery is functionally equivalent to movement preparation. For instance, when demands of the locomotor tasks require increasing cognitive and sensory information processing, the left SMA becomes progressively engaged (34).

The increased SPL activity for St1 relative to St0 observed from the P_50_ component and strengthened after N_90_, suggests that this region contributed to the sensory facilitation via thalamocortical projections. Indeed, a large proportion of thalamic neurons directly project to the SPL (55–58). The increase of the SPL region could entail that the sensorimotor integration mechanisms were updating the current body representation to adapt the feedforward setting of the APAs as evidenced later with the smaller thrust peak in the Loaded compared to the Control conditions. In the Loaded condition, a crucial update of the body representation was likely needed as loading increases sensory and motor noise (59). This is in line with the proposition that to update body representation, simultaneous integration of sensory and motor signals overtime is required (10, 60). A key region for this process would be the SPL as it has been demonstrated that a patient with a lesion of the SPL failed to maintain a constant grip force or to perform accurate slow reaching movement in absence of vision (61). The authors suggested that, for this patient, the storage mechanism was damaged thus stored state estimate of body representation decayed over time.

During the later stage of the preparation process (St2) the sensory transmission did not remain as high as in the early stage of the preparation phase (St1) likely because at that time the APAs preparation was almost finished thus online change was not possible. For instance, MacKinnon et al. (7) reported that when a startle-like acoustic stimulus was delivered to release the planned movement 100 ms before the go cue signal for step initiation, the muscles activation sequence was like control voluntary step in duration and amplitude. The fact that St2 P_50_-N_90_ magnitude was like St0 (Standing only) confirms that no further down- or up-regulation of somatosensory transmission occurred as it was reported in a previous study (16).

In conclusion, sensory facilitation is restored at an early stage of the preparation process, that is, when participants needed to perform proficient APAs before executing stepping movements. This action occurs regardless of the quantity of afferents arriving at the cortical level. Specifically, when plantar sole cutaneous afferents were attenuated, sensory processing could involve both interconnections between the primary somatosensory cortex and SMA and an indirect thalamic connection to PPC which bypass primary somatosensory cortex. Restoration of sensory facilitation in SPL and SMA regions prior to stepping is consistent with the involvement of these two sensorimotor areas in body representation and motor preparation.

## Author Contributions

OL, NT, MS, and LM contributed to the conception and design of the study, organized the database. OL performed the statistical analysis. OL and LM wrote the first draft of the manuscript. All authors contributed to manuscript revision, read, and approved the submitted version.

### Conflict of Interest Statement

The authors declare that the research was conducted in the absence of any commercial or financial relationships that could be construed as a potential conflict of interest.
